# Adaptation of *Musca domestica* L. Field Population to Laboratory Breeding Causes Transcriptional Alterations

**DOI:** 10.1371/journal.pone.0085965

**Published:** 2014-01-28

**Authors:** Dorte H. Højland, Karl-Martin Vagn Jensen, Michael Kristensen

**Affiliations:** Department of Agroecology, Aarhus University, Aarhus, Denmark; University of Crete, Greece

## Abstract

**Background:**

The housefly, *Musca domestica*, has developed resistance to most insecticides applied for its control. Expression of genes coding for detoxification enzymes play a role in the response of the housefly when encountered by a xenobiotic. The highest level of constitutive gene expression of nine P450 genes was previously found in a newly-collected susceptible field population in comparison to three insecticide-resistant laboratory strains and a laboratory reference strain.

**Results:**

We compared gene expression of five P450s by qPCR as well as global gene expression by RNAseq in the newly-acquired field population (845b) in generation F_1_, F_13_ and F_29_ to test how gene expression changes following laboratory adaption. Four (*CYP6A1*, *CYP6A36*, *CYP6D3*, *CYP6G4*) of five investigated P450 genes adapted to breeding by decreasing expression. *CYP6D1* showed higher female expression in F_29_ than in F_1_. For males, about half of the genes accessed in the global gene expression were up-regulated in F_13_ and F_29_ in comparison with the F_1_ population. In females, 60% of the genes were up-regulated in F_13_ in comparison with F_1_, while 33% were up-regulated in F_29_. Forty potential P450 genes were identified. In most cases, P450 gene expression was decreased in F_13_ flies in comparison with F_1_. Gene expression then increased from F_13_ to F_29_ in males and decreased further in females.

**Conclusion:**

The global gene expression changes massively during adaptation to laboratory breeding. In general, global expression decreased as a result of laboratory adaption in males, while female expression was not unidirectional. Expression of P450 genes was in general down-regulated as a result of laboratory adaption. Expression of hexamerin, coding for a storage protein was increased, while gene expression of genes coding for amylases decreased. This suggests a major impact of the surrounding environment on gene response to xenobiotics and genetic composition of housefly strains.

## Introduction

The housefly (*Musca domestica* L.) is a highly mobile cosmopolitan pest, which comes into contact with excreta, carcasses, garbage and other septic matter, and is intimately associated with humans, our food and utensils. Thus the housefly is potentially involved in transmission of many serious and widespread diseases such as salmonellosis, typhoid fever, cholera and infantile diarrhea and amoebic dysentery [1], [2]. Despite the fact that the housefly is a passive vector, its activity in husbandry can result in lower levels of milk and egg production in addition to reduced food conversion [3]. Given the importance of houseflies in the transmission of human and animal diseases, effective control of houseflies is essential for limiting the spread of disease and the economic loss associated with lower production.

Houseflies are controlled by pesticides, which on a large scale lead to resistance. Resistance to pesticides is a chronic and widespread problem, associated with almost all types of insecticides and in most cases caused by increased detoxification or reduced binding of the insecticide to the target site [4], [5]. For the efficacy evaluation of insecticides, including resistance risk assessments, bioassays are pivotal. In this context bioassays are performed with an insecticide-susceptible reference laboratory strain and usually a series of resistant laboratory populations as well as field populations [5], [6]. It is only the heterogeneous nature of field populations that allows for the selection of rare variants corresponding to resistance alleles which are likely to trigger control failure [7]. In the field, selection acts on a large population sizes while selection in the laboratory is done with relatively few inbred individuals, creating a bottleneck.

Toxicity of insecticides varies between susceptible field populations and susceptible laboratory strains, as well as between field populations, where large unexplained variations of toxicity of unexposed field populations occur [8]. These differences or natural variation could be referred to as differences in tolerance or sensitivity, whereas the term resistance is best defined as a reduction in susceptibility beyond natural variation, causing control failure [9]. A key element in preventing development of resistance as well as resistance management is the understanding of this natural variation in tolerance to insecticides, which is the foundation of the microevolutionary process leading to or preventing resistance.

In previous studies we elucidate how expression of P450 genes of laboratory-adapted strains relate to expression in field strains (as well as differences in male and female P450 expression patterns), since the xenobiotic response of P450 is known to play an important role in the development of insecticide resistance and possibly also in the general toxicity of insecticides [10], [11]. Included in these studies was a newly-acquired field strain, 845b, which proved to be susceptible to the insecticides spinosad, pyrethroid and imidacloprid to the same extent as most field populations tested in Denmark [10]. Even though 845b was susceptible, the highest level of constitutive gene expression of nine P450 genes was found in this strain compared to a multi-resistant laboratory strain and the susceptible reference strain WHO-SRS [10]. Expression of P450 genes was increased in 845b males and females compared to WHO-SRS in all cases, including 150-fold male *CYP6D3* expression in 845b compared to WHO-SRS. This very high level of P450 gene expression in 845b raised the question: can data from laboratory-adapted strains be related to natural populations? It could be hypothesized that environmental epigenetics is a factor in expression of xenobiotic metabolism genes in the housefly, where heritable changes in gene expression occur without changes in genomic sequence. Laboratory strains will during their adaptation to life in captivity loss the parental imprinting preparing them for a harsh environment or phrased differently: Does gene expression decrease when houseflies are domesticated and how can parental imprinting be restored? This study will serve as a stepping stone in examining the effects of domestication to laboratory breeding on gene expression in a newly-collected housefly strain. We follow the effects to laboratory settings by exploiting the great opportunities of next generation sequencing technology. We compare housefly global gene expression patterns in three groups of houseflies; F_1_ houseflies, F_13_ houseflies (ten months) as well as F_29_ houseflies (21 months) of both sexes. An overview of changes in P450 expression as well as a description of the changes of global gene expression will be given.

## Materials and Methods

### Housefly strains and breeding

The insecticide-susceptible standard reference strain WHO-SRS was received in 1988 from the Department of Animal Biology, University of Pavia, Italy.

The field population 845b was collected in 2011 at a dairy farm located at Salbækvej 50, Flade, Nykøbing Mors, Denmark (56°53′51.07″N, 8°48′42.81″E). The flies were collected on private land with consent of the owner. The field collection did not involve endangered or protected species. It was tested by two discriminating doses of spinosad and imidacloprid in a non-choice feeding bioassay. Resistance to pyrethrin synergized by PBO was tested in a topical application bioassay. The spinosad resistance level of 845b was in the same order of magnitude to what was observed in the 31 populations in our previous study, which were considered spinosad-susceptible [6]. The bioassay with PBO synergized pyrethrin and imidacloprid showed that 845b had a low level of resistance [10]. The strain could be characterized as a normal Danish field population with no or low level of resistance to commonly used insecticides.

Housefly breeding followed standard laboratory conditions. Egg laying was performed on crumpled filter paper soaked in whole milk. Breeding jars (5 L plastic buckets) containing 4 L of medium were seeded with 200 mg of eggs, corresponding to 2700 eggs. The breeding medium consisted of wheat bran 400 g, lucerne meal 200 g, baker's yeast 10 g, malt extract 15 mL, whole milk 500 mL and water 500 mL. For adult feeding, cube sugar and water were given continuously. Feeding started after emergence with whole-milk powder mixed with icing sugar (1∶1 w/w) [11].

### Houseflies for gene expression analysis

Five to seven days old, adult male and female flies were fed sugar coated with acetone as the only food source. Oral application is a secure method of ensuring exposure. This is standard for constitutive gene expression analysis as described in Markussen and Kristensen [12]. This is done to be able to compare these data with possible insectide-treated flies, since they will be fed sugar coated with insecticide dissolved in acetone. All flies had access to water, milk and sugar *ad libitum* before trials. A number of fly batches ranging from 130 to 500 specimens were placed in cages with full access to water and were given excess of granular sugar in a small petri-dish as the only food. The feeding tests were carried out at 25–26°C, 60–65% RH in continuous light. Twenty-four hours upon test start, living and fresh looking flies were collected by vacuum suction, immediately sedated by cold and killed by freezing. The flies were hereafter kept on -80°C until RNA extraction.

### RNA, DNA and primers

Total RNA from whole bodies of pooled flies (approx. 1.2 g equivalent to 60 flies) was extracted using the RNeasy Maxi Kit (Qiagen). Flies were thoroughly ground with liquid nitrogen, a mortar and pestle and otherwise following the manufacturer's protocol. Isolated RNA was DNase-treated and concentrated using the RNeasy MinElute Kit (Qiagen). Gel electrophoresis and spectrophotometry (Nanodrop; NanoDrop Technologies, Wilmington, USA) was performed to assess the integrity and the concentration of each RNA sample, which was dissolved in RNase-free water and stored at −20°C until use.

Extraction of gDNA used for external standards was performed according to the manufacturer's protocol for the DNeasy Kit (Qiagen). Genomic DNA was stored as stocks of 125 ng µL^−1^ at −20°C corresponding to ∼120,000 copies of a single-copy gene. The mass of the haploid housefly genome (the C-value; http://www.genomesize.com) is ∼1.04 pg therefore 1 ng of gDNA from *M. domestica* contains *ca*. 962 copies of a single-copy gene. A fresh 10-fold serial dilution at five quantities ranging from 125 ng (∼120,000 gene copies) to 0.0125 ng (∼12 gene copies) was prepared for each real-time PCR run.

Gene specific primer pairs were designed based on sequences obtained from the NCBI GenBank: *CYP6A1* (M25367), F: 5′-aattttgccaatcgtggtctg-3′, R: 5′-tccaccattaccaagtggcc-3; *CYP6A36* (DQ642009), F: 5′-aaaggcatggccgttgttat-3′, R: 5′-acttgagaagcggcaaaatg-3′; *CYP6D1* (U22366), F: 5′-gcaaatgcactcaggatttcc-3′, R: 5′-tgcccaagagggagatgataa-3′; *CYP6D3* (AF200191), F: 5′-tgccccataagggaggct-3′, R: 5′-agaccattgactggtactaaaaccg-3′;*CYP6G4* (FJ911556), F: 5′-gctgcaaagcaaattggg-3′, R: 5′-actacgcaccacattcag-3′.

The primer pairs used were designed not to span introns since the present study used gDNA for external standards in real-time PCR runs. To avoid non-specific amplification all RNA samples were routinely treated with DNase before use. Upon optimization forward and reverse primers were used in optimal concentration 150 nM. Amplicon sequence specificity was verified by dissociation curves giving rise to single peaks at the specific melting temperature of the products.

### RT reaction and real-time PCR

First-strand cDNA was synthesized from RNA followed by PCR using 150 nM of primers specific for the *CYP6A1*, *CYP6A36*, *CYP6D1*, *CYP6D3* and *CYP6G4* genes as described by Markussen and Kristensen [12]. All samples and the external standards were run in four replicates per run. Each sample was run multiple times. These four replicates of each sample indicates the measurement precision, whereas the strain variance is accounted for by randomization of the flies selected for RNA purification, two to four biological replicas as well as the number of flies used; approx. 60 houseflies per sample.

The PCR runs were performed on ABI PRISM 7500 HT Sequence Detection Systems with Sequence Detection system software version 1.4 (ABI) initiated by a 2 min activation step at 50°C followed by a polymerase activation step for 10 min at 95°C. Amplification was obtained by 40 cycles of 15 s at 95°C with a 1 min anneal and extending step at 60°C. A final dissociation stage at 95°C for 15 sec, 60°C for 15 sec and 95°C for 15 sec was added to generate a melting curve for verification of amplification product specificity. The qPCR data are presented as the mean copy number per 20 ng of RNA ± standard deviation of minimum four replicates. Statistical analysis for qPCR data was undertaken using a pairwise Wilcoxon non-parametric test, where a P-value less than 0.05 was considered to be statistically significant (SAS, version 9.3). Statistical analysis for overall expression from transcriptome data was undertaken using a Paired t-test, where a P-value less than 0.05 was considered to be statistically significant (R: A Language and Environment for Statistical Computing, R Foundation, 2012).

### Preparation of housefly transcriptome

For the identification of transcripts in the global expression experiment a normalized cDNA library was prepared from 12.2 µg mRNA prepared from adult male and female houseflies. From the total RNA sample poly(A)+ RNA was isolated, which was used for cDNA synthesis. First-strand cDNA synthesis was primed with a N6 randomized primer. Then 454 adapters were ligated to the 5′ and 3′ ends of the cDNA. The cDNA was finally amplified with PCR (15 cycles) using a proof reading enzyme. Normalization was carried out by one cycle of denaturation and re-association of the cDNA. After hydroxylapatite chromatography, the ss-cDNA was PCR amplified (6 cycles).

The normalized cDNA library was size fractioned to approx. 500–1,200 bp. High throughput sequencing on GS FLX++ of the *Musca* cDNA library was done according to the standard protocols using a Genome Sequencer FLX Titanium Instrument (Roche Diagnostics). We got 666,442 reads (316,904,800 bases in total) with the maximum single read length of 1,123 bp and the max modal read length was 518 bp and mean length was 475 bp. Clustering and assembly of all reads in contigs after the sequencing were done using MIRA 4.0 and contigs were initially analyzed by BLAST analysis. Preparation of cDNA, normalization and sequencing was performed by Eurofins MWG GmbH (Ebersberg, Germany).

### Gene expression quantification by RNAseq

For comparison of gene expression eight 3′-fragment cDNA libraries was prepared by standard polyA-tailed priming, cDNA synthesis, gel sizing, PCR amplification, library purification and quality control. Non-normalized cDNA libraries were prepared from a) 1.9 µg RNA from male 845b generation 1 (F_1_), b) 4.7 µg RNA from female 845b (F_1_), c) 5.3 µg RNA from male 845b generation 13 (F_13_), d) 5.4 µg RNA from female 845b (F_13_), e) 4.8 µg RNA from male 845b generation 29 (F_29_), f) 4.8 µg RNA from female 845b (F_29_), g) 4.2 µg RNA from male WHO-SRS, h) 2.0 µg RNA from female WHO-SRS.

Quantification of the eight cDNA libraries was carried out on a HiSeq 2000 v3.0 Genome Analyzer (Illumnia Inc.) by producing 100 bp single-end fragment sequences. The yield of the eight samples ranged from 1,451 Mb to 2,422 Mb. A total data set of 14,136 Mb was filtered for quality and sorted according to the contig index created by the above *Musca* transcriptome. The expression data were normalized to glyceraldehyde 3-phosphate dehydrogenase (GAPDH).

Preparation of cDNA, sequencing and initial indexing was performed by Eurofins MWG GmbH (Ebersberg, Germany).

## Results

The hypothesis: “Gene expression will decrease with time during domestication (laboratory breeding) of houseflies” was established based on prior investigation of P450 expression, where the F_1_ generation of a field collected population showed extraordinarily high level of expression [10]. Initially this hypothesis was followed by repeating expression experiments by quantitative PCR in later generations (F_13_ and F_29_), but to be able to get a more general statement about gene expression alterations following adaptation to breeding in the laboratory, a RNAseq experiment elucidating the global expression pattern of the three generations was performed. Quantitative PCR is performed with gene specific primers, and multiple replicas are performed in order to obtain reliable results. It can be a time-consuming process, where relatively large amounts of RNA are needed. Problems with qPCR might include reproducibility, true sensitivity and specificity, but can discriminate between closely related mRNAs [13]. The transcriptome method is a sample-of-one method, which has the advantage that small amounts of RNA are needed for a successful analysis. Transcriptome analysis has become a valuable alternative to the more time-consuming qPCR, but it is still limited by the extensive bioinformatics skills required by the biologist for proper data analysis [14].

### *CYP6A1* gene expression

When accessing qPCR data, gene expression of *CYP6A1* was significantly higher in the F_1_ generation of the 845b strain, compared to later generations of houseflies (Table 1). Gene expression decreased 6.6-fold and 10.7-fold for males and females, respectively in the F_13_ population (P value_male_: <0.0001, P value_female_: <0.0001), but no further decrease was shown after F_13_ in males (P value_male_: 0.0683), but female expression further decreased (P value_female_: 0.0002).

**Table 1:** Constitutive P450 gene expression of the housefly field strain 845b over 21 months of laboratory adaption measured by quantitative real-time PCR.

Gene	Generation		Male			Female	
		n	copy number	ranking	n	Copy number	ranking
***CYP6A1***	F_1_	32	44.6±6.41	a	33	21.6±9.62	a
	F_13_	38	6.81±4.95	b	38	2.01±0.83	b
	F_29_	23	5.25±4.31	b	14	2.91±0.73	c
***CYP6A36***	F_1_	19	84.5±27.9	a	31	57.5±23.5	a
	F_13_	17	10.4±3.02	b	23	8.12±2.61	b
	F_29_	15	8.81±6.48	b	15	11.7±2.00	c
***CYP6D1***	F_1_	20	1,793±582	a	39	824±446	a
	F_13_	26	657±328	b	27	553±284	b
	F_29_	26	1,045±655	b	15	1,147±202	c
***CYP6D3***	F_1_	32	739±237	a	57	241±162	a
	F_13_	41	169±73.7	b	41	129±72.7	b
	F_29_	40	255±182	b	28	192±39.2	a
***CYP6G4***	F_1_	29	513±87.4	a	48	203±142	a
	F_13_	38	141±80.5	b	43	138±82.1	b
	F_29_	44	273±201	c	28	150±75.8	ab

Mean mRNA transcript copy number ×1000 is per 20 ng of total RNA. Ranking of significance levels (5%) between comparisons of fly generations were assigned a, b and c, to indicate significance.

**Figure 1:**
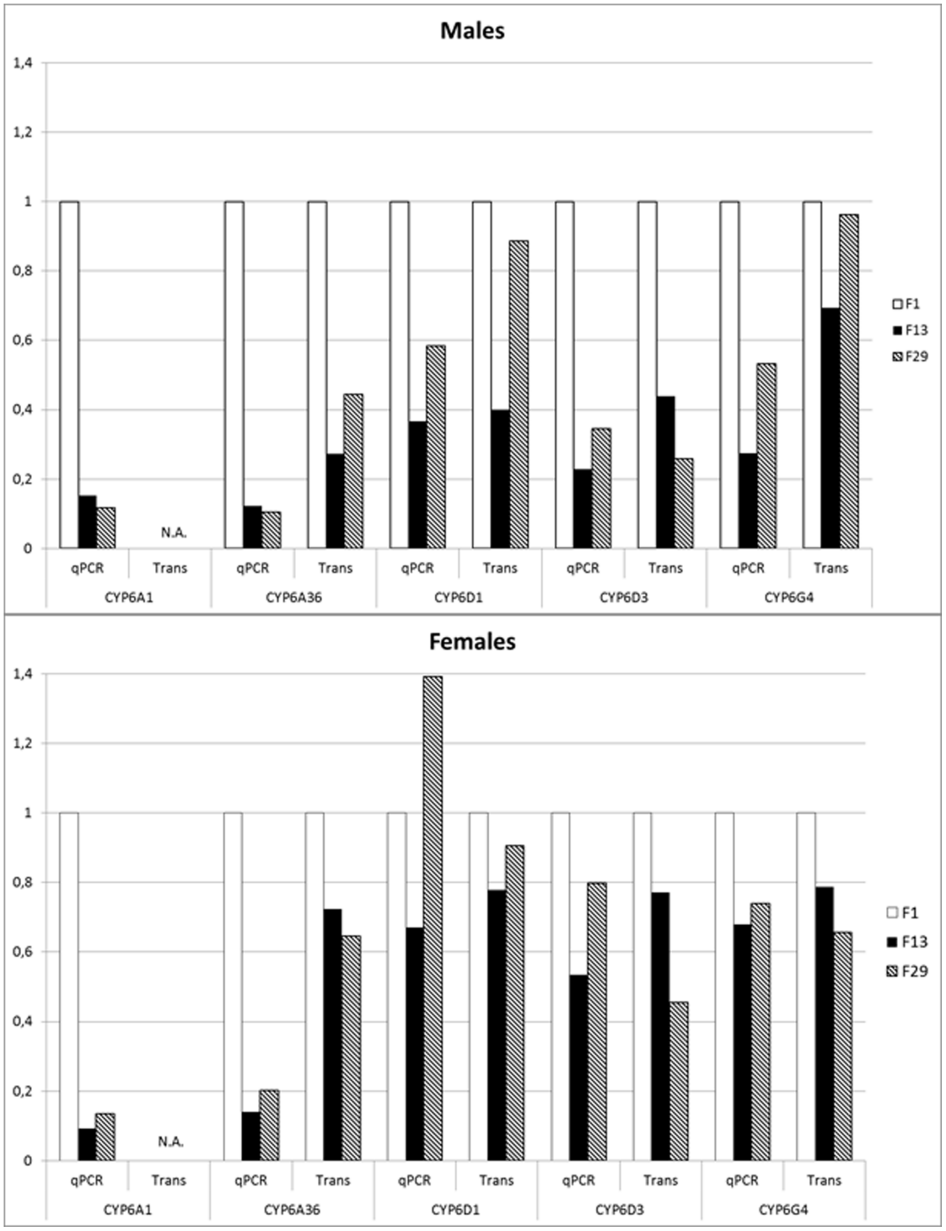
P450 gene expression over time using qPCR and transcriptome analysis in 845b males and females. Data is normalized to gene expression for F_1_ males and females, respectively. Copies of *CYP6A1* were not observed in the transcriptome analysis, and *CYP6A1* is denoted not applicable. Trans: transcriptome data.

According to the transcriptome data, only one and three transcript of *CYP6A1* was present in F_1_ males and F_13_ females, respectively. For the remaining groups, no sequences representing *CYP6A1* were found (Figure 1) and the data can't be used for assessment of this apparently lowly expressed gene. Large variances in the qPCR data was observed in all three generation groups, but in the F_1_ generation distribution of data points was much wider than later in the adaption process (F_13_ and F_29_ flies), where the variance within the samples decreased, which left data points in distinct groups significantly different from each other rather than overlap due to large sample variances.

### *CYP6A36* gene expression

The gene expression pattern observed for *CYP6A36* using qPCR is similar to that of *CYP6A1* with decreasing expression over time (Table 1). A similar pattern was observed for the transcriptome data, where gene expression decreased over time. For male flies, the overall variance of the sample changed over time from approx. 10% in F_1_ flies to 25% in F_29_ flies, while gene expression decreased more than 8-fold (P value: <0.0001). For females, on the other hand variances within samples decreased over time, while gene expression decreased 7-fold and 5-fold, respectively. Both F_13_ and F_29_ houseflies had a significantly lower level of *CYP6A36* gene expression than the F_1_ flies for both males and females (P value_F1–F13_: <0.0001, P value_F1–F29_: <0.0001). No further decrease in *CYP6A36* gene expression was observed between F_13_ and F_29_ flies in males (P value_male_: 0.5209), but a decrease was observed in females (P value_female_: 0.0003) when analyzing the qPCR data. Minor changes were observed in the transcriptome data.

### *CYP6D1* gene expression

Gene expression of *CYP6D1* showed a different expression pattern than that of the *CYP6A* genes (Table 1). According to qPCR, male *CYP6D1* constitutive gene expression decreased almost 2-fold after 29 generations (P value_F1–F29_: 0.0004), but gene expression of *CYP6D1* was no different in the F_29_ than in F_13_ houseflies (P value_F13–F29_: 0.0582). Female *CYP6D1* gene expression decreased significantly in F_13_ in comparison to F_1_ (P value_F1–F13_: 0.0067), but the level of *CYP6D1* gene expression in F_29_ increased to a level significantly (1.4-fold) higher than in the F_1_ generation (P value_F1–F29_: 0.0120; Table 1) when accessing qPCR data. Female *CYP6D1* gene expression in F_29_ was higher than F_1_ according to qPCR, but was not elevated according to transcriptome data.

### *CYP6D3* gene expression

With both transcriptome and qPCR, male and female, F_1_ houseflies had the highest *CYP6D3* gene expression (Table 1). According to qPCR, expression was decreased 4-fold and 3-fold in F_13_ and F_29_ males (P value_F1–F13_: <0.0001, P value_F1–F29_: <0.0001), respectively when compared to F_1_, but no further difference in gene expression was observed between F_13_ and F_29_ males (P value_F13–F29_: 0.0662). With qPCR, female *CYP6D3* gene expression decreased almost 2-fold in the F_13_ generation (P value_F1–F13_: 0.0006), but increased again after 29 generations of laboratory adaption, to a level equal to both the initial gene expression level of F_1_ female flies (P value_F1–F29_: 0.1486), but different from the F_13_ generation (P value_F13–F29_: 0.0006; Table 1). The transcriptome analysis showed a decrease in *CYP6D3* gene expression as adaption progressed.

### *CYP6G4* gene expression

For the qPCR data, *CYP6G4* gene expression in both male and female houseflies decreased significantly from F_1_ to F_13_ flies (Table 1) in agreement with the transcriptome analysis (Figure 1). Gene expression of *CYP6G4* increased from F_13_ to F_29_ in males. For males, *CYP6G4* gene expression in the F_29_ population was 1.9-fold lower than the F_1_ (P value_F1–F29_: <0.0001), but significantly higher than in F_13_ males (P value_F13–F29_: 0.0241). The female F_29_ flies had a qPCR gene expression level similar to both the F_1_ and F_13_ population (P value_F1–F29_: 0.0648; P value_F13–F29_: 0.5524), despite F_1_ and F_13_ being significantly different from each other (P value_F1–F13_: 0.0348). *CYP6G4* gene expression decreased continuously for females according to the transcriptome data.

### Global gene expression analysis by RNAseq

For comparison of gene expression eight 3′-fragment non-normalized cDNA libraries was prepared. The cDNA libraries were prepared from 845b male and female F_1_, F_13_, F_29_ and WHO-SRS houseflies. Quantification of the eight cDNA libraries was carried out by RNAseq by producing 100 bp single-end fragment sequences (14,136 Mb). The sequencing yield of the eight samples was: F_1_ male 2,422 Mb, F_1_ female 1,913 Mb, F_13_ male 1,921 Mb, F_13_ female 1,451 Mb, F_29_ male 1,640 Mb, F_29_ female 1,447 Mb, WHO-SRS male 1,748 Mb and WHO-SRS female 1,594 Mb. These primary data were clustered in contigs and compared to the annotated *Musca* transcriptome (see Materials and methods for details). The full data set is available as Table S1. The expression data were normalized to glyceraldehyde 3-phosphate dehydrogenase (GAPDH). The level of gene expression were compared between adult male and female houseflies in the three generations and WHO-SRS was included as a fully domesticated strain, which has been in breeding for >1,200 generations. A total of 35,836 contigs were obtained from the analysis. Any contig with less than 10 sequences in the F_1_ populations was eliminated from the data set as ‘noise’, since the effect of randomness was considered to be too high. This modification left 19,755 and 19,150 sequences for males and females, respectively (Table 2).

**Table 2:** Number of genes up-regulated and down-regulated as an effect of laboratory adaption in male and female 845b houseflies.

		Limits	F13		F29	
			Number of genes	% of genes	Number of genes	% of genes
**Males**	**Upregulation**	**≥1.2**	370	2	4,644	24
	**Unchanged**	**0.8–1.2**	700	4	5,630	28
	**Downregulation**	**≤0.8**	18,683	94	9,479	48
**Females**	**Upregulation**	**≥1.2**	4,899	26	8,410	44
	**Unchanged**	**0.8–1.2**	5,934	31	4,842	25
	**Downregulation**	**≤0.8**	8,314	43	5,895	31

Sequences which were found in less than ten copies in F_1_ flies were considered background noise. This left 19,756 and 19,150 sequences for males and females, respectively. Values of F_13_ and F_29_ above 1.2-fold F_1_ fly expression were considered up-regulated, while values below 0.8-fold F_1_ were considered down-regulated. Values above 0.8-fold and below 1.2-fold were characterized as ‘unchanged’ from the F_1_ flies.

Analysis of male gene expression showed that almost all genes were down-regulated in F_13_ in comparison with the F_1_ population, while 24% and 28% were up-regulated and down-regulated in F_29_, respectively. In females, 26% of the approximately 19,000 genes were up-regulated in F_13_ in comparison with F_1_, while 43% were down-regulated. Equal numbers of genes were up-regulated, down-regulated and unchanged from F_1_ to F_29_ in females (Table 2).

**Figure 2:**
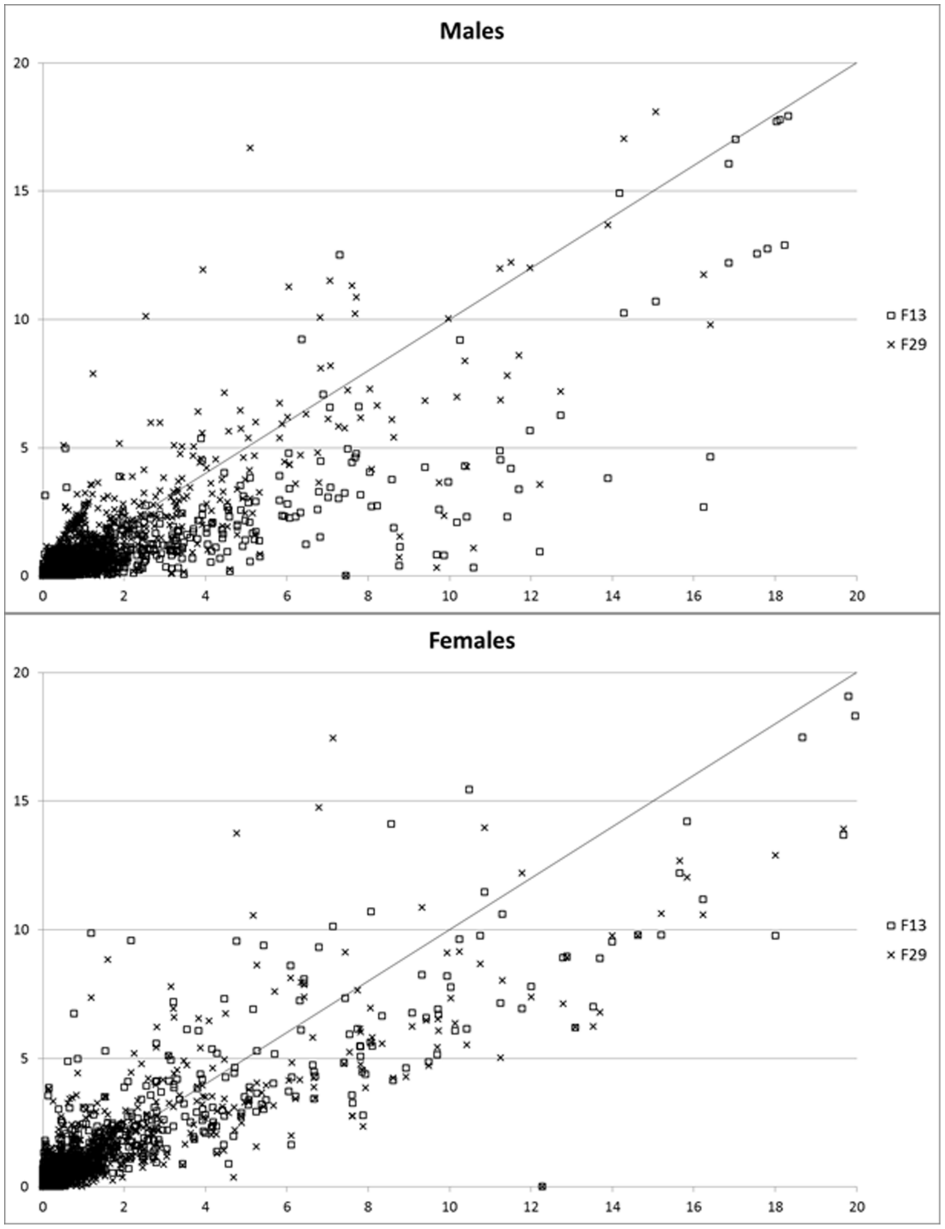
Total gene expression of 845b F_13_ and F_29_ male and female as a function of the F_1_ gene expression. The line represents no difference from the F_1_ flies. Square represents F_13_ and cross represents F_29_. Genes in right-lower and left-upper corner are down-regulated and up-regulated over time, respectively. A few genes surpassed 20 and were omitted from the figure.

When assessing the dataset as three time points (Figure 2) with each 19,755 observations (males) and 19,150 observations (females), the overall expression of genes were down-regulated between F_1_ and F_13_ males (P value_F1–F13_: <0.0001), while F_29_ males had a significant higher expression level than F_13_ (P value_F13–F29_: <0.0001), but lower than F_1_ (P value_F1–F29_: 0.0053). In females, gene expression in F_29_ was significantly higher than F_13_ (P value_F13–F29_: <0.0001), which in turn was significantly higher than F_1_ gene expression (P value_F1–F13_: <0.0001).

### Expression of P450 genes

An initial search of the annotated 454-transcriptome contigs identified 86 potential P450 genes showing either similarity to *M. domestica* P450s or to other insect P450 present in GenBank. Further analysis by comparison and alignment of these sequences led to the 40 P450s presented in Table 3. Most of the housefly P450s currently available at GenBank was identified, with a noteworthy exception of *CYP12A1*, which we have included in our earlier investigations.

**Table 3:** Constitutive P450 gene expression of the housefly field strain 845b and reference strain WHO-SRS over 21 months of laboratory adaption measured by RNAseq.

P450	Transcriptome contig	GenBank annotation	F1 M	F13 M	F29 M	WHO-SRS M	F1 F	F13 F	F29 F	WHO-SRS F
**GAPDH**	**c14446**	**AY675185**	**1,000 (3,040)**	**1,000 (6,450)**	**1,000 (3,240)**	**1,000 (2,820)**	**1,000 (3,440)**	**1,000 (3,410)**	**1,000 (3,110)**	**1,000 (2,850)**
*CYP4D3*	c146	EF615000	117	83	146	97	387	424	192	**244**
*CYP4D4*	c1971	EF615001	79	25	54	**31**	113	100	73	**48**
*CYP4D35*	c21960	DQ642007	6	2	2	**3**	5	4	2	**3**
*CYP4G2*	c1956, c6971, c7817, c11288, c11387, c22714, c22765	EF615002	2,771	813	2,224	**1,747**	9,890	3,482	4,073	**7,953**
*CYP4G13*	c6003, c13456, c17283, c17586, c30318	AF355145	18,371	3,248	13,600	**6,970**	72	592	85	**3,948**
*CYP6A1* a	c19753	M25367	0	0	0	**0**	1	0	0	**0**
*CYP6A4*	c23657	U09232	0	1	1	**3**	1	1	0	**1**
*CYP6A5*	c9709	EF615004	3	0	1	**1**	6	2	1	**1**
*CYP6A24*	c7163	AB050019	28	6	2	**0**	19	15	5	**0**
*CYP6A25*	c3948, c25525	AF240401	10	8	12	**60**	29	21	16	**40**
*CYP6A36* a	c17998	DQ642009	65	8	27	**43**	38	28	27	**28**
*CYP6A37*	c29, c35216	DQ642010	153	69	116	**118**	92	117	72	**45**
*CYP6A38*	c34193	EF615003	706	201	108	**8**	398	464	235	**7**
*CYP6A40*	c11673	FJ911555	7	1	2	**9**	2	0	3	**3**
*CYP6C2*	c1227	U09345	0	0	1	**1**	1	1	0	**1**
*CYP6D1* a	c5625, c14635	U22366	1,645	310	1,369	**709**	1,004	788	1,007	**365**
*CYP6D3* a	c6762, c12837	AF285767	562	116	136	**155**	200	156	101	**40**
*CYP6D8*	c4916, c12096	FJ911557	435	108	201	**333**	231	247	208	**62**
*CYP6G4* a	c6373, c13526	FJ911556	1,318	431	1,192	**830**	979	777	711	**333**
*CYP12A2*	c9347, c26394	U94698	116	24	83	**147**	61	44	42	**43**
*CYP12A3*	c4957, c23288	U94699	149	39	133	**211**	140	89	80	**82**
*CYP28B1*	c1608, c32995	AF355144	917	252	543	**398**	477	431	333	**97**
**P450-like genes**		**Similar to:**								
*CYP4*-like	c4622	*B. dorsalis* P450; HQ257450	123	73	57	**140**	70	99	42	**73**
*CYP4*-like	c8304	*B. dorsalis* P450 CYP4; GU292424	18	8	17	**16**	29	20	17	**9**
*CYP4D*-like	c19345	*M. domestica* CYP4D4; EF615001	26	4	19	**40**	16	22	17	**15**
*CYP6A*-like	c9873	*M. domestica* CYP6A5; EF615004	17	5	10	**9**	8	16	8	**2**
*CYP6*-like	c19497	*D. melanogaster* CYP6V1; NM_134559	33	11	51	**46**	38	38	33	**56**
*CYP6*-like	c17048	*G. morsitans* CYP6U1; EZ422519	11	2	2	**16**	3	1	1	**19**
*CYP6*-like	c18509	*L. cuprina* CYP6A27; DQ917666	16	2	8	**0**	12	7	2	**0**
*CYP9*-like	c7313	*D. melanogaster* CYP9F2; NM_141932	186	89	218	**232**	273	212	192	**91**
*CYP12*-like	c4504	*D. melanogaster* CYP12E1; NM_141746	742	201	305	**16**	1,012	435	381	**14**
*CYP12A*-like	c5039	*M. domestica* CYP12A3; U94699	18	6	9	**47**	14	23	11	**23**
*CYP12A*-like	c26961	*M. domestica* CYP12A3; U94699	13	7	11	**16**	13	26	11	**10**
*CYP28B*-like	c21570	*M. domestica* CYP28B1; AF355144	14	3	14	**7**	11	11	8	**6**
*CYP28*-like	c247	*C. capitata* P450 28d1-like; XM_004519855	121	141	229	**630**	85	343	217	**141**
*CYP302*-like	c20782	*C. capitata* P450 302A1; XM_004525241	2	1	3	**7**	3	2	4	**8**
*CYP304*-like	c4527	*C. capitata* P450 304A1-like; XM_004521269	62	29	25	**52**	75	27	57	**25**
*CYP308*-like	c2674, c7833	*C. capitata* P450 308A1-like; XM_004536721	135	161	240	**362**	266	733	267	**340**
*CYP313*-like	c21453	*D. melanogaster* P450 CYP313B1; NM_141550	1	1	1	**2**	2	1	3	**2**
P450	c673	*G. morsitans* P450; EZ423604	2	2	2	**14**	1	4	1	**5**

Contig name, annotation and number of copies of P450 and P450-like genes in 845b and WHO-SRS males and females. Data were normalized to glyceraldehyde 3-phosphate dehydrogenase (GAPDH). *B. dorsalis*: *Bactrocera dorsalis*, *D. melanogaster: Drosophila melanogaster, M. domestica: Musca domestica, G. morsitans: Glossina morsitans, L. cuprina: Lucilia cuprina, C. capitata: Ceratitis capitata*.

a Genes also analysed by qPCR.

When looking through the data set, 22 P450 genes were found and 18 groups of P450-like genes (Table 3). In most cases, P450 gene expression was decreased in F_13_ flies in comparison with F_1_ for both males and females. Gene expression then increased from F_13_ to F_29_ in males and decreased further in females. A few genes showed no change in gene expression over time. These include *CYP6A4*, *CYP6A25* and *CYP6C2* in both sexes. *CYP6A40* and *CYP6D8* both remained unchanged in females, but decreased in males over time. For the P450-like genes, most of those were either down-regulated or unchanged over time and in most cases with the highest copy number for F_1_ flies (Table 3).

### Expression of other genes

To exemplify the global transcription data presented above, which is a very broad view of the houseflies gene expression, various genes were selected for more detailed description (Table 4) – to elucidate how RNAseq data like these can be used for expression analysis.

**Table 4:** Constitutive gene expression of the housefly field strain 845b and reference strain WHO-SRS over 21 months of laboratory adaption measured by RNAseq.

Gene	Transcriptome contig	GenBank annotation	F1 M	F13 M	F29 M	WHO-SRS M	F1 F	F13 F	F29 F	WHO-SRS F
**GAPDH**	**c14446**	**AY675185**	**1,000 (3,040)**	**1,000 (6,450)**	**1,000 (3,240)**	**1,000 (2,820)**	**1,000 (3,440)**	**1,000 (3,410)**	**1,000 (3,110)**	**1,000 (2,850)**
Superoxide dismutase, SOD	6618	AY460107	925	386	808	**763**	1,160	982	638	**1,020**
Superoxide dismutase, SOD1	c533	JF919738	150	16	86	**171**	83	15	80	**163**
Superoxide dismutase, SOD2	c5897	JF919739	460	275	466	**701**	640	497	449	**641**
Attacin 1	c7768, c7461	AY460106, DQ062744, AY725024	2,200	143	259	**835**	578	173	17	**462**
Attacin 2	c8314, c12003, c14832, c15082, c15680, c20356, c20368, c35230	FJ794603	2,920	203	255	**273**	268	324	4	**209**
Hexamerin F1	c16955	AY256681	73	3,130	169	**31**	772	6,720	2,330	**82**
Hexamerin F3	c17278	AF188888	30	399	20	**66**	886	2,030	2,440	**164**
Yolk protein 1	c5690, c13879, c23274, c7345, c14388, c14365, c6070, c29622	X97008	8	18	1	**80**	1,690	1,910	4,500	**345**
Yolk protein 2	c5544, c32461, c13516, c21667,	X97009	0	8	1	**2**	2,820	958	3,310	**425**
Yolk protein 3	c29795	X97010	13	173	103	**29**	1,970	1,930	3,650	**160**
Alpha-amylase	c5526, c14492, c33368, c16610	EF494036	24,100	3,490	6,820	**32,600**	11,600	6,320	5,950	**4,220**
Alpha-tubulin	C5871, c12028	Similar to *C.eratitis capitata* alpha-tubulin; XM_004519499	5,050	1,660	5,370	**6,400**	4,330	4,670	4,690	**7,810**
Beta-tubulin	c5846	Similar to *Glossina. morsitans* beta-tubulin; DQ377071	8,050	4,030	7,280	**7,540**	6,100	8,590	8,130	**14,600**
Actin	c15480	Similar to *Chrysomya megacephala* actin; KC207081	3,440	2,450	5,030	**4,810**	2,810	4,110	6,200	**1,830**
Ribosomal protein L15	c5862	Similar to *Drosophila virilis* ribosomal L15 protein; DQ426903	10,400	4,290	8,390	**11,500**	19,700	13,700	13,900	**49,000**
40S ribosomal protein S26	c6954	Similar to *Glossina morsitans* 40S ribosomal protein S26; EZ424337	12,000	5,670	12,000	**16,400**	21,300	19,300	19,400	**47,600**
Ribosomal protein S25	c765	Similar to *Drosophila melanogaster* ribosomal protein S25; NM_169376	7,820	3,160	6,150	**7,220**	16,200	11,200	10,600	**35,200**
Ribosomal protein L36	c143	Similar to *Stomoxys calcitrans r*ibosomal protein L36; EZ048838	5,060	2,840	5,380	**7,210**	14,000	9,530	9,770	**31,000**

Contig name, annotation and number of copies of diverse groups of *Musca domestica* genes in 845b and WHO-SRS males and females. Data are normalized to GAPDH (the number in parenthesis signifies the actual observed number for GAPDH).

Several forms of superoxide dismutase (SOD; an enzyme important for the antioxidant defense and also linked to the xenobiotic response [15]) were observed in the transcriptome data set, all of which were decreased over time to various degrees. When combining the numbers for all SOD forms found, a clear decrease was observed between F_1_ and F_13_ in males (2.2-fold) and between F_1_ and F_29_ in females (1.6-fold).

Gene expression of the antibacterial peptide, attacin, which is part of the non-specific insect immune system [16], decreased 15-fold and 10-fold in F_13_ and F_29_ males in comparison with F_1_, respectively. In females, a 40-fold decrease was observed between F_1_ and F_29_, while expression decreased <2-fold in F_13._

The overall expression of genes encoding the storage protein hexamerin [17] increased 1.8-fold and 2.9-fold over time in males and females, respectively.

Yolk protein was included in this study as a female-specific protein [18]. Indeed, gene expression of genes coding for yolk protein was much higher in females than in males (Table 4). Female gene expression of these genes changed >1.8-fold, while males gene expression was increased 5-fold in F_29_ compared to F_1_.

In male F_1_ houseflies, expression of alpha-amylase, which hydrolyses alpha bonds of large, alpha-linked polysaccharides [19], was similar to that of WHO-SRS (Table 4), but decreased 3.5-fold after 29 generations. A <2-fold decrease was observed in females, causing gene expression in F_13_ and F_29_ to be similar to that of WHO-SRS.

Genes coding for tubulin and actin were included in this list due to their potential as reference genes similar to GAPDH. Their expression was not altered more than 2.2-fold in both directions.

Ribosomes are composed of ribosomal RNA molecules and a variety of proteins making up the translational apparatus. The ribosomal proteins are potentially interesting since their abundance might reflect translational activity [20]. Here, we follow the expression of four ribosomal protein genes (Table 4). Gene expression in F_1_ females proved higher than in F_13_ and F_29_, while F_1_ males had 2-fold higher expression than F_13_ flies, but was not different from F_29_ males.

## Discussion

We compared gene expression profiles of more than 19,000 genes, with special focus on five cytochrome P450 genes of the *CYP6* family with relation to detoxification of insecticides in a Danish housefly field strain. This was done at three time points over the course of 29 generations (equal to 21 months) of laboratory adaption. The five genes have previously been shown to have an extraordinary high gene expression in 845b F_1_ population in comparison to laboratory adapted strains [10]. We compared results gained from qPCR and transcriptome analysis. Analysis by transcriptome is a fast and efficient alternative to the more time-consuming qPCR. But transcriptome analysis used as a gene expression tool demands considerations about the depth of the analysis, bearing in mind the lack of *CYP12A1* copies and low level of expression of *CYP6A1* detected by RNAseq compared to qPCR.

The overall transcriptome data set included 35,836 sequences. The highest gene expression observed for F_1_ males and females represented a parasite (the protist *Oxytricha trifallax*), which indicate that the F_1_ flies were infected when captured. Infections are not uncommon in field flies, and as adaption continues in the laboratory pathogens will be eliminated. These genes were excluded from the analysis, and are not data set presented here. In general, global gene expression was decreased over time in males, given the limits set in Table 2. A higher proportion of genes were up-regulated in females compared to males over time, but the majority of genes were still down-regulated in F_13_ compared to F_1_ females. However, the same proportion of genes was up-regulated in F_29_ compared to F_1_ (Table 2).

The transcriptome analysis was performed to possibly validate the patterns observed for five P450s using qPCR in prior work [10]. The qPCR method is widely used to evaluate gene expression in different samples. When comparing data from the two experiments, similar patterns were observed. In most cases, both methods showed a decline in gene expression over time (Figure 1). In both analytical methods *CYP6A1* was lowly expressed in comparison with the other CYPs, but in this study, *CYP6A1* was only represented with one copy in the transcriptome data set, making it useless for any conclusions. The transcriptome data otherwise supports the qPCR results, suggesting that detoxification P450 genes are indeed down-regulated as a result of adaption to laboratory breeding.

Male constitutive expression of three genes (*CYP6A1*, *CYP6D1* and *CYP6D3*), which was shown to be extraordinarily highly expressed in 845b F_1_ flies compared to three laboratory adapted strains by Højland *et al*. [10], were all decreased significantly after 29 generations of laboratory adaption. *CYP6A1* is possibly linked to organochlorine and organophosphate-resistance [21], while *CYP6D1* and *CYP6D3* has been linked to pyrethroid-resistance [21], [22]. The decrease in expression of these three genes suggests that they are more important in the wild than in a laboratory setting.

*CYP6A36* has, like *CYP6D1*, been associated with pyrethroid resistance in the USA [23], [24]. Much like *CYP6A1*, *CYP6A36* gene expression was decreased over time in 845b.

*CYP6G4* is a possible ortholog of the *CYP6G1* gene in *D. melanogaster* and constitutive overexpression of *CYP6G1* is important in DDT and neonicotinoid resistance in the fruit fly [25], [26]. Recently, *CYP6G4* has shown to be over-expressed in a pyrethroid-resistant housefly strain from China [27]. Here, *CYP6G4* gene expression decreased significantly in males by qPCR analysis, while expression in F_29_ and F_1_ were similar in the transcriptome data set. Female *CYP6G4* gene expression on the other hand was not significantly different between the adapted F_29_ flies and the other two time points.

What causes P450 gene expression in some cases, to remain at the same level, or even increase after 21 months of laboratory adaption is unknown, but it could be speculated whether some houseflies still hang on to some of their defensive responses to toxins, inherited from their wild ancestors. Gene expression of the five P450s, related to detoxification of xenobiotics, decreased significantly from the initial F_1_ generation to F_13_ flies, which has been adapting to the laboratory for approx. ten months. In most cases, gene expression did not change further from F_13_ flies to F_29_ flies suggesting a relatively fast adaption to new surroundings and environmental pressure. Maintaining a constantly alert detoxification system is very demanding in terms of energy, so if it is not needed, it will most likely be “turned off” or at least down-prioritized [28].

In general, the qPCR data set caused large deviations overall. The large variances in 845b flies of the F_1_ generation could be caused by the fact that these flies are ‘fresh’ from the field, causing the data to represent the actually variance present in field populations. As the flies adapt to laboratory conditions, one might expect the data to become more grouped, indicating the creation of a more unified population. As laboratory adaption progressed, the qPCR data did become more grouped. Unfortunately, in most cases these groups proved significantly different from each other, thus the large variances were not eliminated.

SOD is one the components protecting the organism from oxidative stress, and is an indicator for the general stress condition of an organism. Here, significant differences in expression of SOD genes were only observed between F_1_ and F_13_, and F_1_ and F_29_ in males and females, respectively. However, expression in F_13_ and F_29_ were generally lower than in F_1_, indicating that houseflies are less stressed in a laboratory setting than in the field.

Genes for the antibacterial peptide, attacin, was significantly decreased over time. This suggests that the flies were infected with a bacterial infection when first captured. It is assumed to be common for houseflies in the field to threatened by bacterial infections practically living in a sea of pathogens, and as they adapt to laboratory conditions without pathogens, they are less threatened and might get more energy to fight off the infection. Therefore less expression of antibacterial genes would be necessary.

Gene expression of the storage protein hexamerin increased when 845b flies were transferred from the field to laboratory breeding, especially in the F_13_ generation. This indicates that storage proteins are important initially after introduction to laboratory settings. Assumable, the food supply is more constant in the laboratory, and energy requirements less than in the field, so storage of energy in case of bad times is increased.

Yolk protein is important in the development of eggs and is associated with females. The data obtained here does also show a higher expression of these genes in females compared with males (which could use it as a storage protein). Gene expression of genes coding for the yolk protein remains unchanged after laboratory adaption, which suggest that development of eggs are not affected by the surrounding environment, but is a fundamental function of female houseflies.

The enzyme alpha-amylase hydrolyses alpha bonds of large, alpha-linked polysaccharides, such as starch and glycogen, yielding glucose and maltose. Dietary carbohydrates are important macromolecules for houseflies and their changed expression of alpha-amylase possibly reflect the adaptation to laboratory food consisting of sugar (sucrose) and protein *ad libitum*.

The ribosomal proteins are potentially interesting since their abundance might reflect translational activity. Decreased expression of ribosomal protein genes could indicate this.

This is our first step in elucidating and understanding the effects of laboratory adaption of housefly field strains. We found that genes, previously shown to be highly expressed in a ‘fresh’ housefly strain, decreased P450 expression as a result of adaption to a laboratory setting when applying the same analytic method as well as transcriptome analysis. Due to the high P450 gene expression in 845b in comparison to laboratory-adapted strains, effects of adaption were tested here. It would be interesting to investigate whether the P450 gene expression decrease observed in 845b here is a general trend in other housefly field strains or whether effects on gene expression of insecticide resistance-related genes only occur in this particular strain. It would be beneficial to test more field strains over a longer time period.

## Supporting Information

Table S1 **Total gene expression of housefly male and female flies from the susceptible strain WHO-SRS, and three generations of field population 845b F1, F13 and F29.**
(XLS)
